# Pan-cancer analysis combined with experiments predicts NNMT as a therapeutic target for human cancers

**DOI:** 10.1007/s12672-024-01052-7

**Published:** 2024-05-29

**Authors:** Hua Huang, Lianchun Su, Ruihao Zhang, Di Wu, Chen Ding, Chen Chen, Guangsheng Zhu, Peijun Cao, Xuanguang Li, Yongwen Li, Hongyu Liu, Jun Chen

**Affiliations:** 1 Department of Lung Cancer Surgery, Tianjin Medical University General Hospital, Tianjin, China; 2 Department of Thoracic Surgery, First Affiliated Hospital of Shihezi University School of Medicine, Shihezi, China; 3 Tianjin Key Laboratory of Lung Cancer Metastasis and Tumor Microenvironment, Tianjin Lung Cancer Institute, Tianjin Medical University General Hospital, Tianjin, China

**Keywords:** Pan-cancer analysis, Bioinformatics, Nicotinamide N-methyltransferase, Tumor immune microenvironment, Immune checkpoint

## Abstract

The identification of effective therapeutic targets plays a pivotal role in advancing cancer treatment outcomes. We employed a comprehensive pan-cancer analysis, complemented by experimental validation, to explore the potential of Nicotinamide N-methyltransferase (NNMT) as a promising therapeutic strategy for human cancers. By analyzing large-scale transcriptomic datasets across various cancer types, we consistently observed upregulated expression of NNMT. Furthermore, elevated NNMT expression correlated with inferior overall survival in multiple cancer cohorts, underscoring its significance as a prognostic biomarker. Additionally, we investigated the relationship between NNMT expression and the tumor immune microenvironment, which plays a crucial role in regulating anti-tumor immune responses. To confirm the malignant functions of NNMT in tumor cells, we conducted a series of cell-based experiments, revealing that NNMT promotes cancer cell proliferation and invasion, indicative of its oncogenic properties. The integration of computational analysis and experimental validation in our study firmly establishes NNMT as a potential therapeutic target for human cancers. Specifically, targeting NNMT holds promise for the development of innovative and effective cancer treatments. Further investigations into NNMT's role in cancer pathogenesis could potentially pave the way for groundbreaking advancements in cancer treatment.

## Introduction

Cancer is a global health burden, so novel therapeutic targets are needed to promote outcomes [1, 2]. In recent years, powerful pan-cancer analyses have emerged, integrating high-throughput transcriptomic data to identify shared molecular alterations across multiple cancer types [3]. These analyses facilitate the identification of potential therapeutic targets applicable to diverse cancers.

A critical metabolic enzyme, Nicotinamide N-methyltransferase (NNMT), plays a significant role in tumors, catalyzing the conversion between nicotinamide and the methyl donor S-adenosylmethionine, resulting in nicotinamide methyl ester and S-adenosylhomocysteine [4]. The overexpression of NNMT has been widely observed in various tumor types, correlating with tumor development and progression [5–7]. NNMT performs multiple functions in tumor cells, enhancing their metabolic adaptability, promoting cell proliferation, and survival. It exerts regulatory control over key signaling pathways, including PI3K/Akt and Wnt/β-catenin, which further promote tumor cell proliferation and invasiveness [8]. Inhibiting NNMT activity or expression holds promise as a potential therapeutic approach to interfere with tumor growth and metastasis [9]. Understanding role of NNMT in tumors provides valuable insights into tumor biology and may contribute to the development of novel anti-cancer therapies [10, 11]. However, a comprehensive pan-cancer analysis, integrating genomic datasets to systematically investigate NNMT dysregulation and its functional implications across various cancers, is currently lacking.

The immune system plays a crucial role in tumor surveillance, and the interplay between cancer cells and immune cells within the tumor microenvironment influences disease progression and therapeutic response [12]. Immune checkpoint molecules, such as programmed cell death protein 1 (PD-1), programmed death-ligand 1 (PD-L1), cytotoxic T-lymphocyte-associated protein 4 (CTLA-4), and T-cell immunoglobulin and mucin domain-containing protein 3 (TIM-3), play pivotal roles in regulating anti-tumor immune responses [13, 14]. Investigating the relationship between NNMT and immune checkpoints could shed light on immunomodulatory functions of NNMT and potential implications for immunotherapy [15]. However, the precise relationship between NNMT and tumor immunity remains unclear. Understanding the tumor immune microenvironment has become critical for developing effective cancer therapies.

Overall, this study combines pan-cancer analysis with experimental validation to explore NNMT as a potential therapeutic targeting in human cancers. This investigation may yield insights into role of NNMT in cancer progression and its implications for therapeutic strategies.

## Materials and methods

### Data sources and processing

NNMT expression was assessed in 26 different tumors and their corresponding normal tissues using The Cancer Genome Atlas (TCGA) cohorts. The SangerBox web-based tool (http://sangerbox.com/) facilitated this analysis. Additionally, NNMT expression levels in different pathological stages (stages I–IV) of selected TCGA tumors were examined through the “Pathological Stage Plot” module of Gene Expression Profiling Interactive Analysis [16]. Violin plots were generated to illustrate the relationship between NNMT expression levels and pathological stages. Furthermore, protein expression levels were investigated using the UALCAN portal (http://ualcan.path.uab.edu/index.html) [17], which provides an interactive web resource for Clinical Proteomic Tumor Analysis Consortium data. The protocol was approved by Ethics Committee of Tianjin Medical University General Hospital.

### NNMT-related gene enrichment analysis

To conduct gene enrichment analysis related to NNMT, we utilized the STRING website (available at https://string-db.org/) [18]. Moreover, the “Similar Gene Detection” module of GEPIA2 identified the top 100 NNMT-associated genes in TCGA tumors. Subsequently, the correlation analysis module of GEPIA2 explored the correlation between NNMT and these top five NNMT-associated targeting genes. Pathway and process enrichment analysis for the identified top 100 NNMT-associated targeting genes was performed using the Metascape web-based tool [19] with specific parameters set to P < 0.01, a minimum count of three for the terms, and an enrichment factor > 1.5 for canonical pathways.

### Analysis of tumor immune and immunosuppressive cell infiltration

The TIMER2 [20] server was used to examine the correlation between NNMT expression and the infiltration of various immune cell types. To assess the effect of genetic and epigenetic alterations of NNMT on dysfunctional T-cell phenotypes, we utilized the QUERY module of the Tumor Immune Dysfunction and Exclusion (TIDE) algorithm [21].

### Epigenetic methylation analysis

The TCGA methylation module within the UALCAN interactive web resource was employed to investigate differences in NNMT methylation levels between tumor and paired normal tissues across various TCGA cancer types [22, 23]. Additionally, the TIDE server was utilized to investigate the effect of NNMT methylation on dysfunctional T-cell phenotypes and prognoses.

### Analysis of gene expression correlations with therapeutic responses

To evaluate the therapeutic potential of NNMT as a target in various cancers, we analyzed drug sensitivity data obtained from the ROC Plotter (http://www.rocplot.org/) [24]. The ROC Plotter is a transcriptome-based tool that enables the prediction of biomarkers by establishing connections between gene expressions and responses to therapy among cancer patients.

### Cell culture and transfection

Human cancer cell lines representing various cancer types, including OVCAR3, HEP3B, PANC1, MCF-7, U87, A549, and human embryonic kidney 293 T cells, were obtained from the American Type Culture Collection (ATCC). The cells were cultured in appropriate media supplemented with 10% fetal bovine serum (FBS). Lentiviral infection was utilized to investigate the impact of NNMT overexpression or knockdown on cellular processes. Lentiviruses for NNMT overexpression, knockdown, and the vector control were procured from GeneChem (China). Following lentiviral infection, stable A549 or U87 cells were established, and puromycin dihydrochloride (Amresco, USA) at a concentration of 4 µg/mL was administered 72 h post-infection to select for successfully infected cells.

### Real-time polymerase chain reaction (PCR)

Total RNA was extracted from transfected cells using a commercially available RNA extraction kit (Sparkjade, Shandong, China) following the manufacturer’s instructions. The concentration and purity of RNA were determined using a spectrophotometer (Takara, Beijing, China). Reverse transcription was performed to synthesize complementary DNA (cDNA), and quantitative real-time polymerase chain reaction (qRT-PCR) was conducted using a reverse transcription kit (Takara, Beijing, China). The relative expression levels of NNMT were normalized to the expression of housekeeping genes, such as GAPDH, and calculated using the 2^-ΔΔCt method. The primer sequences for mRNA were as follows: 5′-GGAGCGAGATCCCTCCAAAAT-3′ and 5′- GGCTGTTGTCATACTTCTCATGG -3′ for GAPDH; 5′-ATATTCTGCCTAGACGGTGTGA-3′ and 5′- TCAGTGACGACGATCTCCTTAAA-3′ for NNMT.

### Cell proliferation assay

Cell proliferation was assessed using the Cell Counting Kit-8 (CCK8) and 5-ethynyl-2'-deoxyuridine (EdU) assay. Cells were seeded into 96-well plates and incubated for 24, 48, and 72 h. At each time point, 10 μL of CCK8 solution was added to each well and incubated for 2 h. The absorbance was measured at 450 nm using a microplate reader. For the EdU assay, the cells were stained with the cell-LightTM EdU Apollo567 In Vitro Kit (Ribobio, Guangzhou, China) following the manufacturer’s instructions. Images were captured using a fluorescence microscope, and the percentage of EdU-positive cells was calculated using Image J.

### Transwell assay

Cell invasion was evaluated using Transwell chambers coated with Matrigel. Cells were seeded into the upper chamber with a serum-free medium, while the lower chamber was filled with a medium containing 10% fetal bovine serum. After incubation for 24 h, the cells that migrated to the lower chamber were fixed with 4% paraformaldehyde and stained with crystal violet.

### Protein extraction and western blot analysis

Total protein was extracted, and protein concentration was measured by the BCA method. The proteins were separated with 8% sodium dodecyl sulfate–polyacrylamide gel electrophoresis, transferred to a polyvinylidene fluoride membrane, and incubated overnight at 4 °C with primary antibodies, including anti-GAPDH (60004-1-Ig, Proteintech) and anti-NNMT (15123-1-AP, Proteintech). Then, the membrane was incubated with anti-rabbit/mouse IgG secondary antibodies (Abclonal, anti-rabbit: AS014, anti-mouse: AS003) at room temperature for 1 h, and bands were developed on the membrane using Syngene G-Box and GeneSnap software (Syngene, Cambridge, UK).

### Statistical analysis

Statistical analysis was performed using GraphPad Prism 8 software. Student’s t-test was used to compare the expression levels of NNMT between different groups, and the Wilcoxon rank-sum test was employed to analyze non-normally distributed data. Pearson correlation analysis was used to evaluate the correlation between NNMT expression and immune infiltration.

## Results

### NNMT localization and expression profiles

**Fig. 1:**
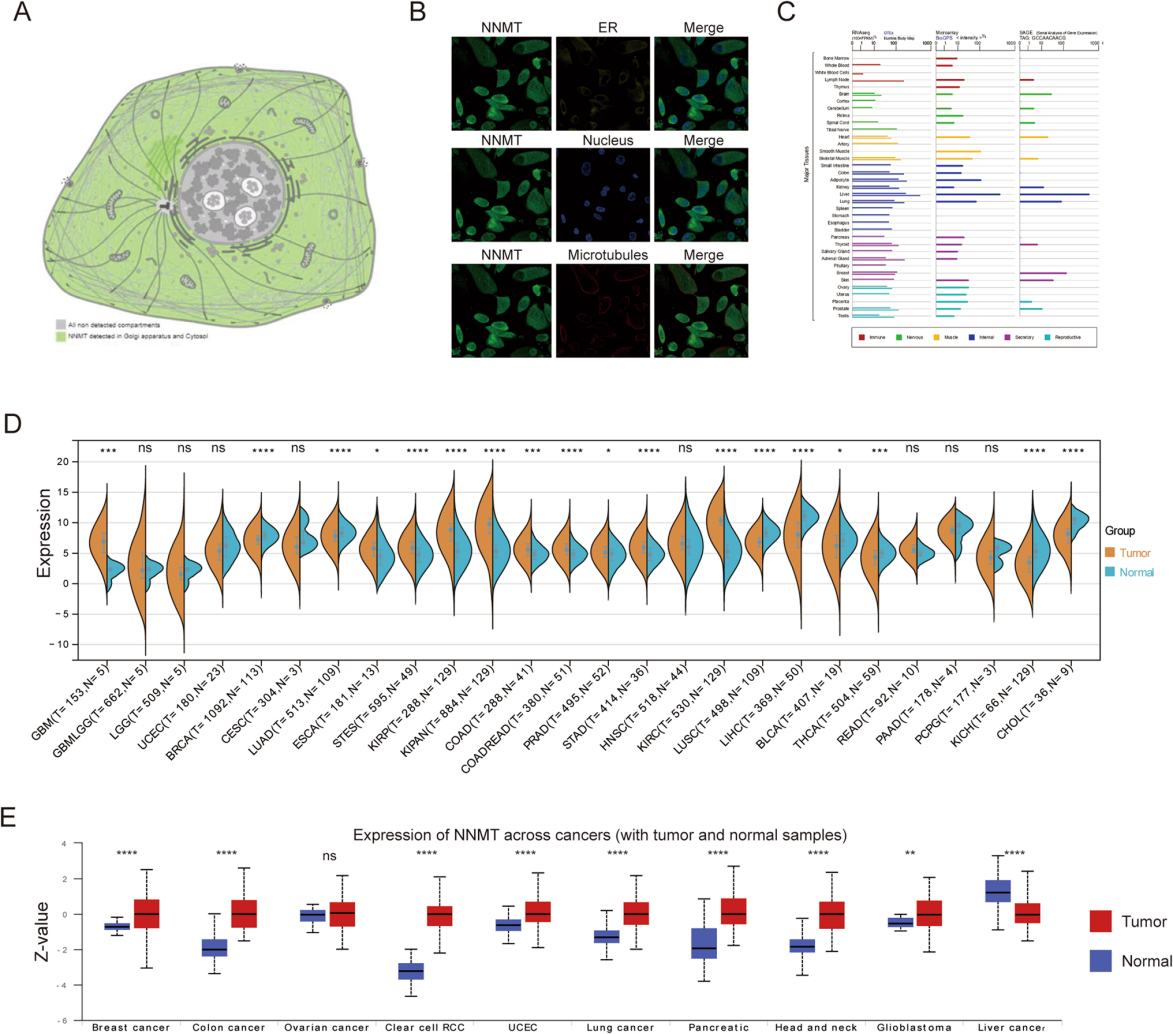
NNMT localization and expression profiles. **A** Cellular localization of NNMT protein in the cytoplasm. **B** Immunofluorescence showing NNMT protein in the cytoplasm of cancer cells. **C** Bar plot presenting NNMT mRNA expressions in various normal human tissues from the GTEx database. **D** NNMT mRNA expression levels in different cancer types based on TCGA data. **E** Protein expression level of NNMT across pan-cancer samples; (nsP > 0.05, *P < 0.05, **P < 0.01, ***P < 0.001, ****P < 0.0001)

NNMT localizes to the cytoplasm in human cells (Fig. 1A). Immunofluorescence assays indicated NNMT's subcellular localization in the cytoplasm, as it colocalized with plasmic markers in cancer cells (Fig. 1B). Additionally, we examined NNMT messenger (m)RNA expression in various normal human tissues, encompassing immune, internal, nervous system, secretory, muscle, and reproductive tissues (Fig. 1C). To comprehensively analyze NNMT expression across multiple cancer types, we conducted a pan-cancer analysis. The findings revealed significant overexpression of NNMT in various cancer types compared to normal tissues (Fig. 1D). Specifically, higher protein expression levels of NNMT were observed in most tumors, including breast cancer, colorectal cancer, clear cell RCC, lung cancer, and several others (Fig. 1E). These results suggest the potential oncogenic role of NNMT in a wide range of cancers.

### NNMT expression is upregulated in various malignant tumours and correlates with shorter survival

**Fig. 2:**
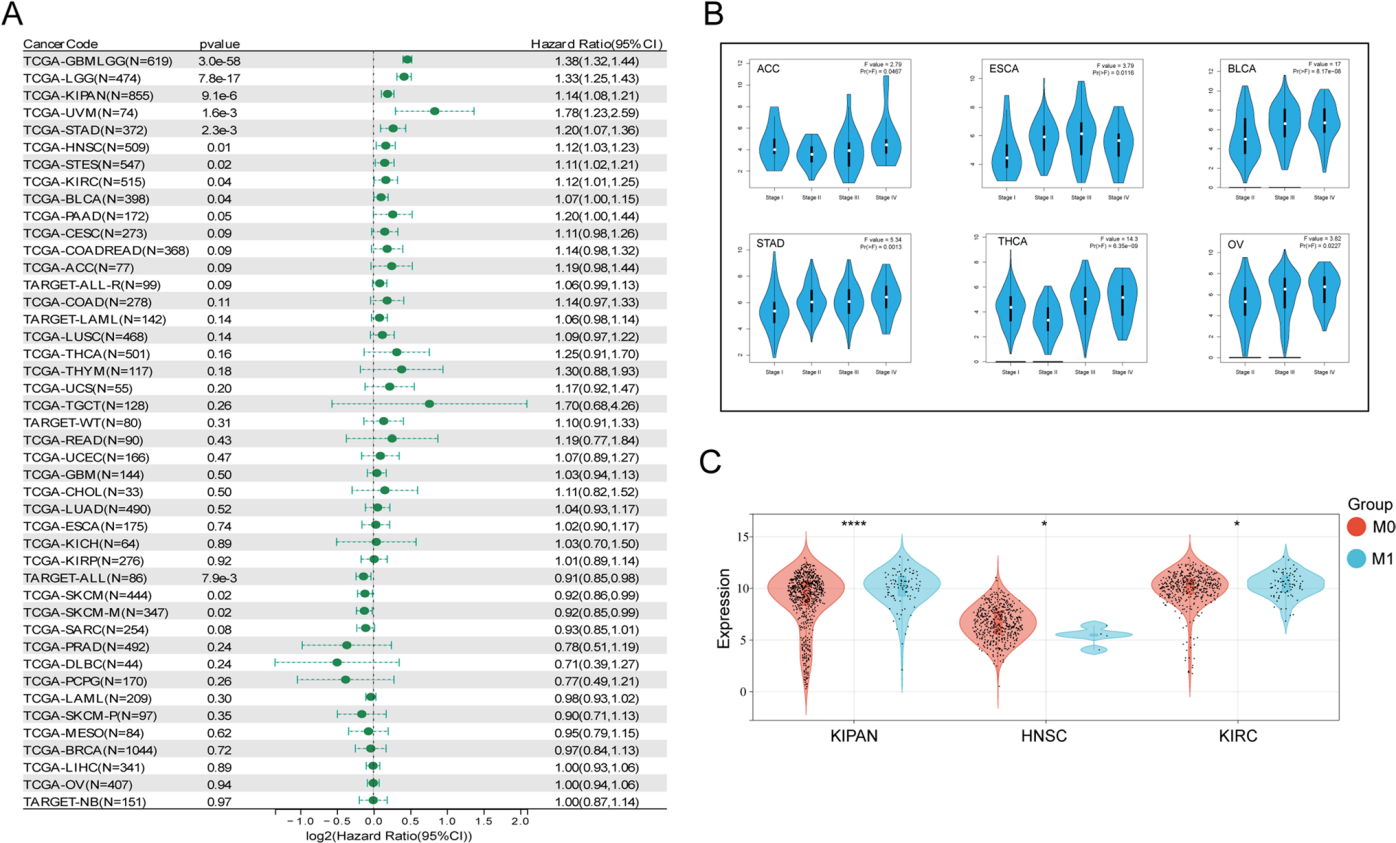
Correlation of NNMT Expression with Prognosis and Clinical Stages. **A** Prognostic association of NNMT expression in various types of cancer using SangerBox. **B** Association of NNMT gene expression levels with pathological stages. **C** Relationship of NNMT expression with metastasis; (*P < 0.05, ****P < 0.0001)

To explore the clinical significance of NNMT expression, we performed survival analysis using large-scale cancer datasets. Consistently, high NNMT expression was associated with poorer prognosis in several cancer types, such as GBMLGG, LGG, KIPAN, UVM, STAD, HNSC, STES, KIRC, BLCA, PAAD (Fig. 2A). Patients with elevated NNMT expression exhibited significantly shorter overall survival OS compared to those with lower NNMT expression levels. Comparing expression profiles between tumor stages, we observed increased NNMT expression at higher tumor stages in ACC, ESCA, BLCA, STAD, OV, THCA (Fig. 2B). Moreover, metastatic KIPAN and KIRC showed higher NNMT expression levels than the corresponding primary tumors, while metastatic HNSC exhibited lower NNMT expression levels (Fig. 2C). These findings indicate that NNMT expression may serve as a prognostic biomarker for cancer patients, albeit in a cancer-type-specific manner, as it varies between different cancer types, suggesting its potential utility in guiding prognosis and treatment decisions tailored to specific cancer contexts.

### DNA methylation analysis of NNMT in *Pan*-*cancer*

**Fig. 3:**
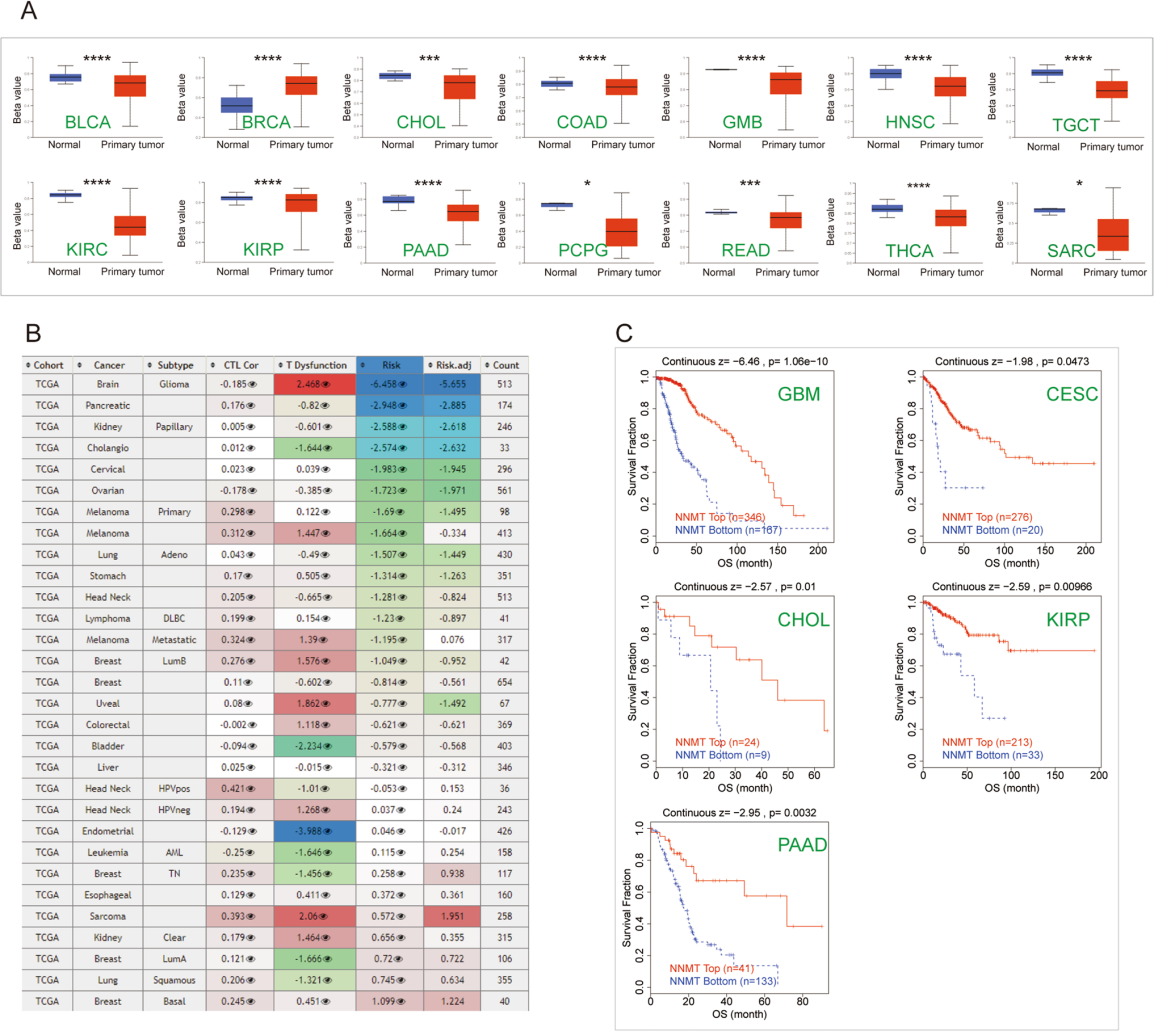
Epigenetic Methylation Analysis (**A**) Boxplots illustrating the differential NNMT methylation levels (beta values) across TCGA cohorts. **B** Heatmap displaying the impact of NNMT methylation on cytotoxic T-cell levels (CTLs), dysfunctional T-cell phenotypes, and risk factors within TCGA cohorts. **C** Kaplan–Meier curves comparing overall survival differences between high and low NNMT methylation levels. Statistically significant differences between cohorts are depicted. (*P < 0.05, ***P < 0.001, ****P < 0.0001)

Changes in DNA methylation in cancer have been heralded as promising targets for the development of powerful diagnostic, prognostic, and predictive biomarkers [25]. NNMT is a key enzyme in nicotinamide metabolism [6]. Studies have confirmed that nicotinamide affects the cellular methyl pool, thereby affecting DNA and histone methylation and gene expression. Analysis of the promoter methylation status revealed that NNMT is hypomethylated in various cancer types, including BLCA, CHOL, COAD, GBM, HNSC, TGCT, KIRC, KIRP, PAAD, PCPG, READ, THCA, and SARC (Fig. 3A). We then evaluated the consequences of NNMT methylation status in various cancers. Interestingly, hypermethylation of NNMT was negatively associated with the risk of patients and correlated with longer OS in the GBM, CESC, CHOL, and KIRP cohorts (Fig. 3B, C).

### Functional enrichment analysis

**Fig. 4:**
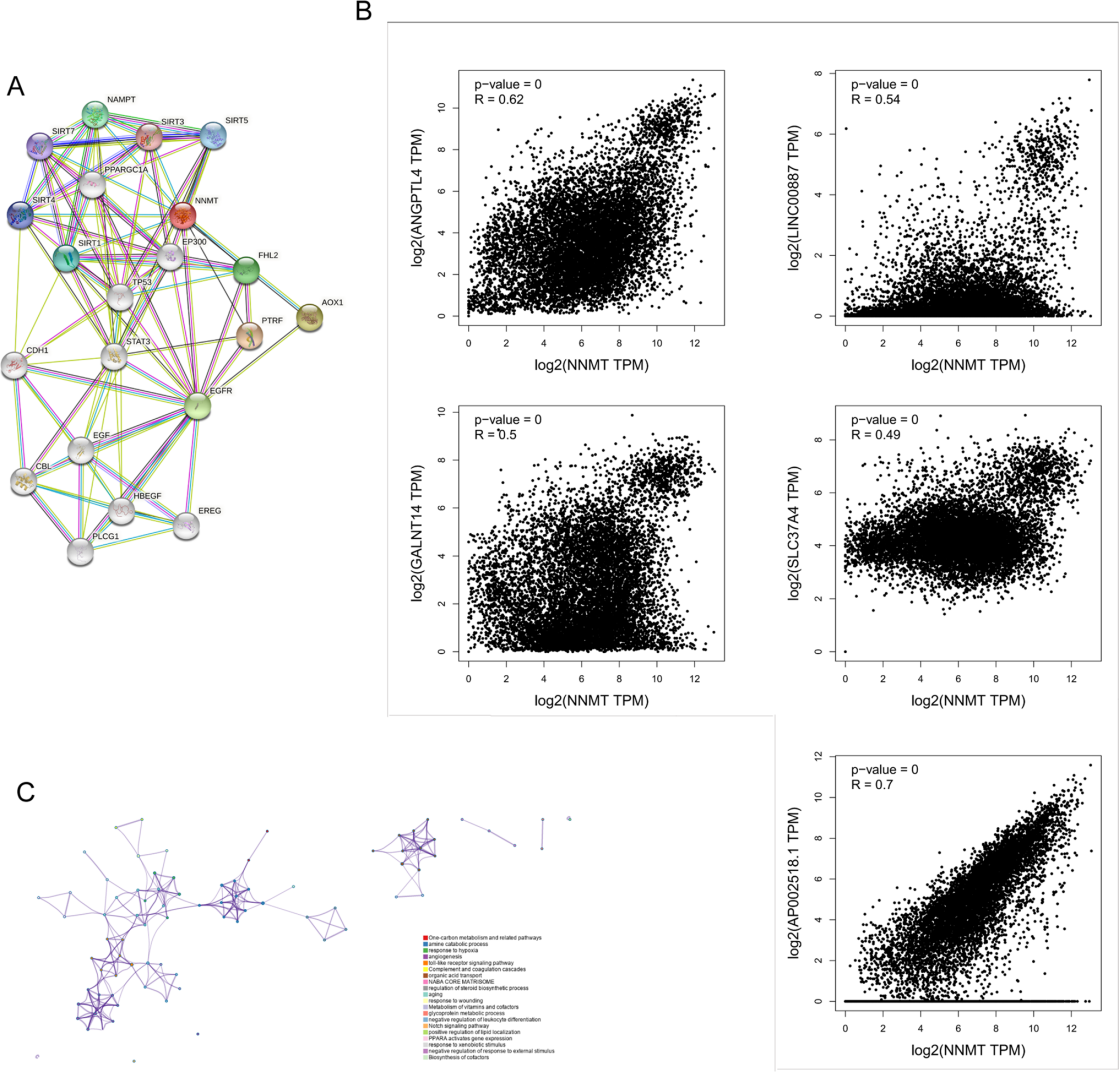
Enrichment Analysis of NNMT-Related Genes across Pan-Cancers. **A** NNMT-binding proteins identified using the STRING tool. **B** The top five NNMT-correlated genes across pan-cancers, and their relationships with NNMT expression analyzed using the GEPIA2 website. **C** Enriched terms with a similarity > 0.3 connected by edges

Using the STRING tool, we screened NNMT-binding proteins to identify its potential role in tumor pathogenesis. The interaction network displayed interactions with TP53, CDH1, STAT3, EGFR, EGF, SIRT3, and others (Fig. 4A). Additionally, correlation and pathway enrichment analysis based on gene expression data from TCGA identified the top five genes significantly correlated with NNMT expression: ANGPTL4, LINC00887, GALNT14, SLC37A4, and AP002518.1 (Fig. 4B). Furthermore, the top 100 NNMT-associated genes were significantly associated with multiple cancer-related signaling pathways, including one-carbon metabolism, amine catabolic process, and response to hypoxia signaling pathway (Fig. 4C). These findings suggest that NNMT may play a role in modulating cancer metabolism.

### NNMT is related to immunity

**Fig. 5:**
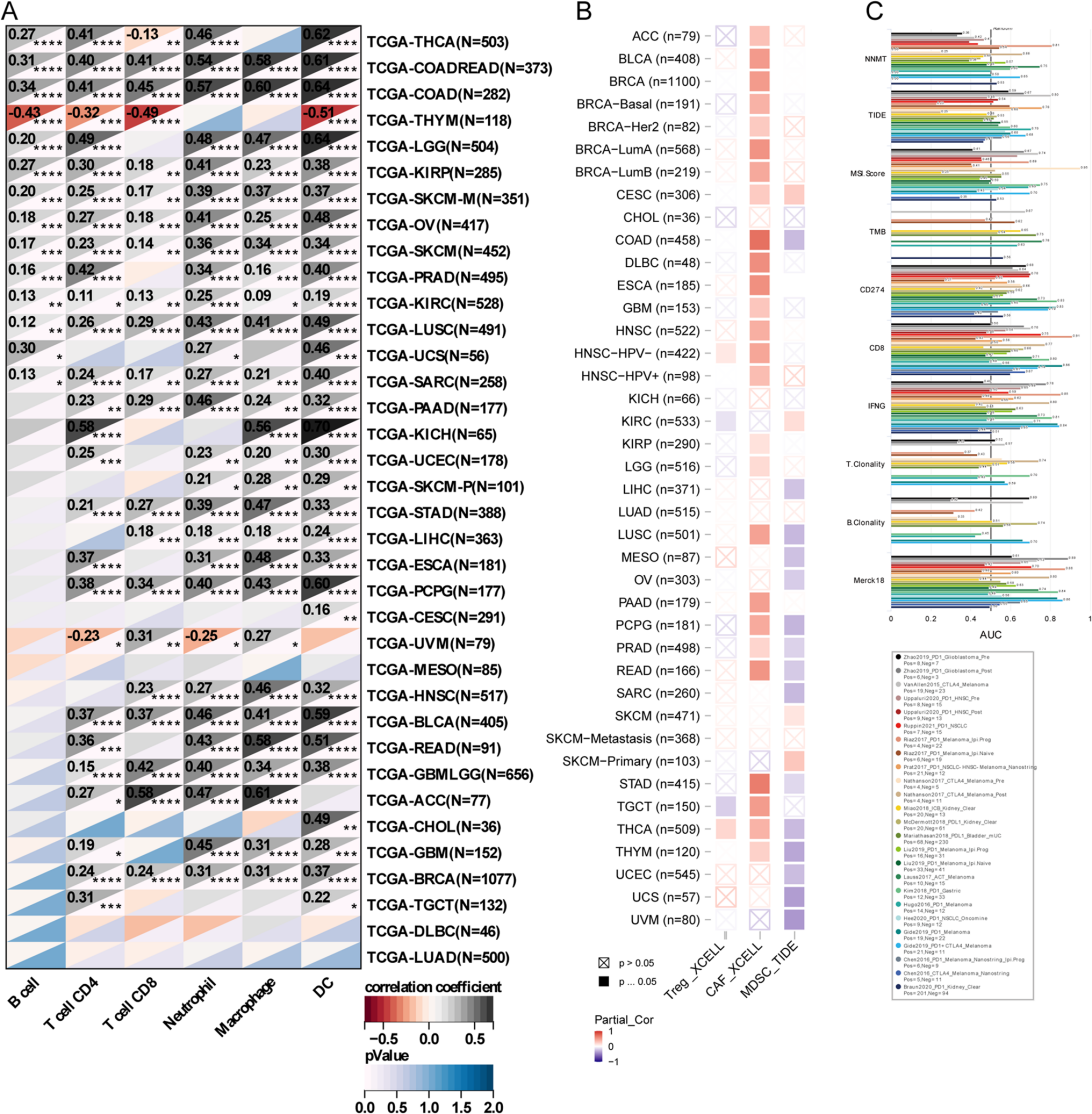
Correlations between NNMT Expression and Immune Cell Infiltration Levels. **A** Heatmap displaying the correlations between NNMT expression and infiltration levels of six immune cell types in TCGA cohorts. **B** Heatmap displaying the correlations between NNMT expression and infiltration levels of three immunosuppressive cell types in TCGA cohorts. **C** A bar plot comparing the biomarker relevance of NNMT with standardized cancer immune evasion biomarkers in immune checkpoint blockade (ICB) subcohorts, including cancer-associated fibroblasts (CAFs), myeloid-derived suppressor cells (MDSCs), and regulatory T cells (Tregs); (nsP > 0.05, *P < 0.05, **P < 0.01, ***P < 0.001, ****P < 0.0001)

Examining the relationship between NNMT expression and immune cell infiltration levels in the tumor microenvironment, we observed a significant positive correlation between NNMT expression and infiltration of six immune cell types (B cells, CD8 + T cells, CD4 + T cells, macrophages, neutrophils, and dendritic cells) in COAD, READ, KIRP, SKCM-M, OV, KIRC, LUSC, and SARC (Fig. 5A). Additionally, we investigated the correlation between NNMT expression levels and infiltration of three immunosuppressive cells that promote T-cell exclusion: myeloid-derived suppressor cells, cancer-associated fibroblasts, and T-regulatory cells. Our findings demonstrated a positive correlation between NNMT expression and tumor infiltration of MDSCs in several cancers, including COAD, LIHC, LUSC, MESO, OV, PCPG, PRAD, READ, SARC, STAD, THCA, THYM, UCEC, UCS, and UVM (Fig. 5B). In most tumors, NNMT expression was positively correlated with the infiltration abundance of Cancer-Associated Fibroblasts (CAF). Furthermore, we compared NNMT with established biomarkers to predict response outcomes and OS of immune checkpoint blockade (ICB) sub-cohorts. The AUC, which measures the predictive accuracy of a biomarker, was calculated for NNMT across 18 ICB sub-cohorts. An AUC value greater than 0.5 indicates predictive power above random chance. Our analysis revealed that NNMT demonstrated an AUC of > 0.5 in 9 out of the 18 ICB sub-cohorts studied. This suggests that NNMT has potential as a predictive biomarker for response outcomes and OS in these specific sub-cohorts, highlighting its utility in guiding treatment decisions in the context of immunotherapy. (Fig. 5C).

### Correlation of NNMT expression with immune checkpoint genes

**Fig. 6:**
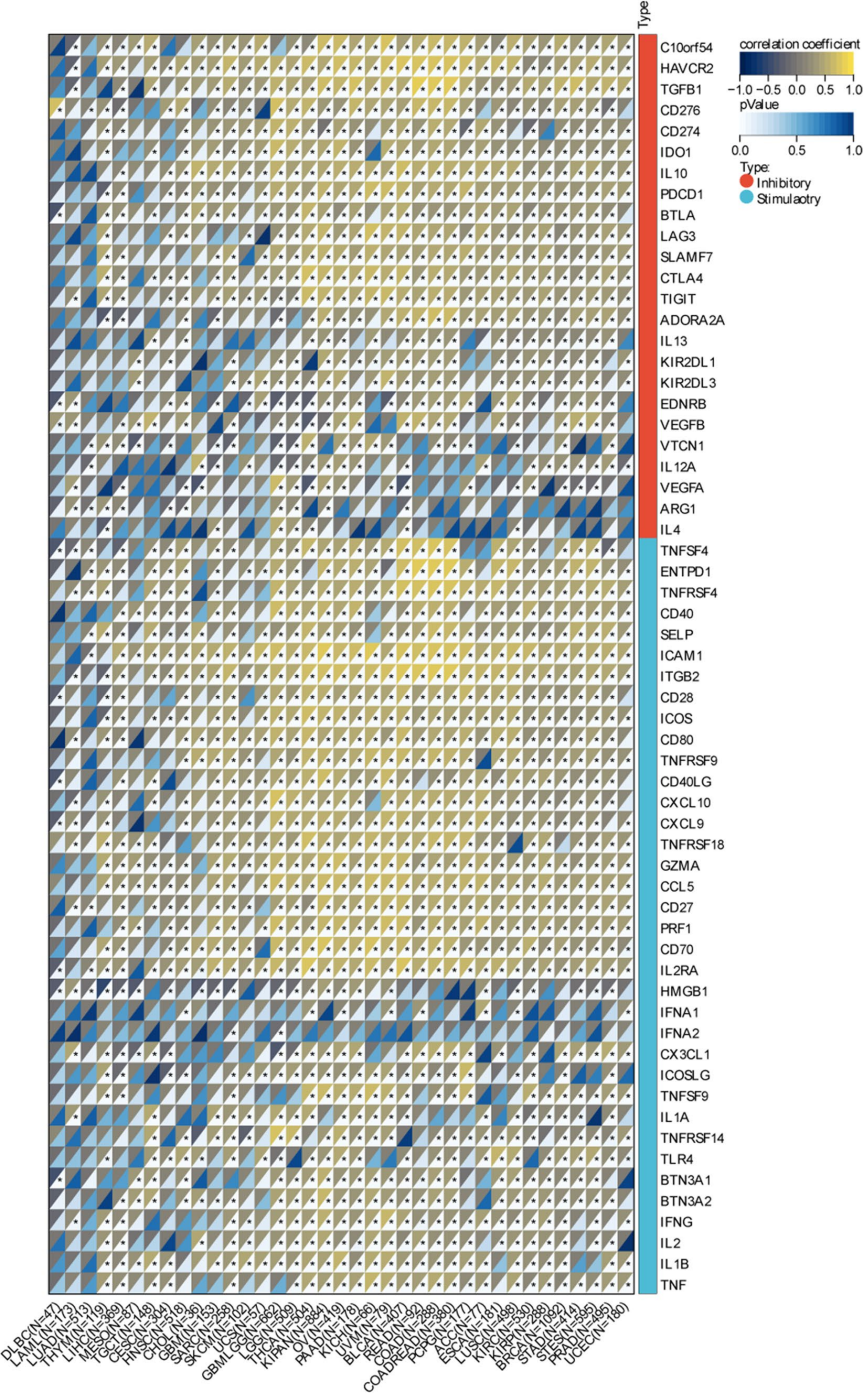
Relationship between NNMT mRNA Expression and Immune Checkpoints in Multiple Cancers; *P < 0.05

We further investigated the correlation between NNMT expression and immune checkpoint genes, critical regulators of immune response in cancer, across 37 cancer types. The expression of NNMT was closely related to the expression of many common immune checkpoints such as PD-L1 and CTLA-4, suggesting that NNMT may be involved in regulating the expression of immune checkpoint genes in cancer (Fig. 6). This finding implies that NNMT may be engaged in immune evasion mechanisms employed by cancer cells through the modulation of immune checkpoint pathways.

### NNMT expression and therapeutic responses

**Fig. 7:**
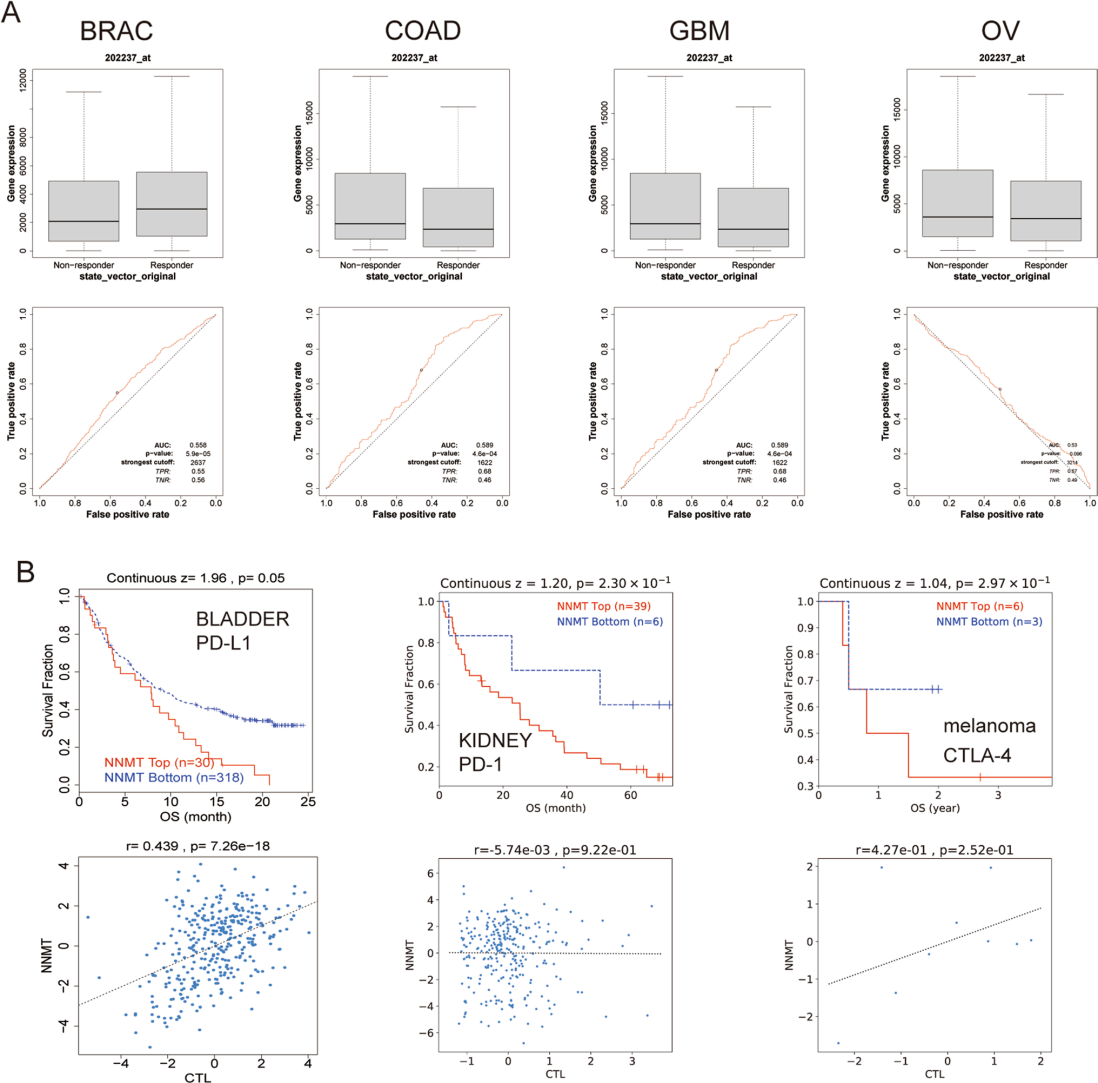
NNMT Expression and Therapeutic Responses in Cancers (**A**) Receiver operating characteristic curve plot of the association between NNMT expression and responses to chemotherapy in cancers. **B** Kaplan–Meier survival curves comparing the immunotherapeutic response in cancers with high and low NNMT expression levels

We examined the associations between NNMT expression and the activities of various clinical chemotherapies in cancer patient cohorts. Our analysis revealed that patients with lower NNMT expression in COAD, GBM, and OV showed increased sensitivity to chemotherapies, while those with higher NNMT expression in BRCA benefited more from such treatments (Fig. 7A). Furthermore, we observed that lower NNMT expression levels were associated with clinical benefits of immune checkpoint blockade (ICB) therapy (PD-1 or PD-L1) in bladder, kidney, and melanoma cancers, leading to prolonged OS compared to cohorts with high NNMT expression levels (Fig. 7B).

### Inhibition of NNMT suppresses cell proliferation and migration in U87 and A549

**Fig. 8:**
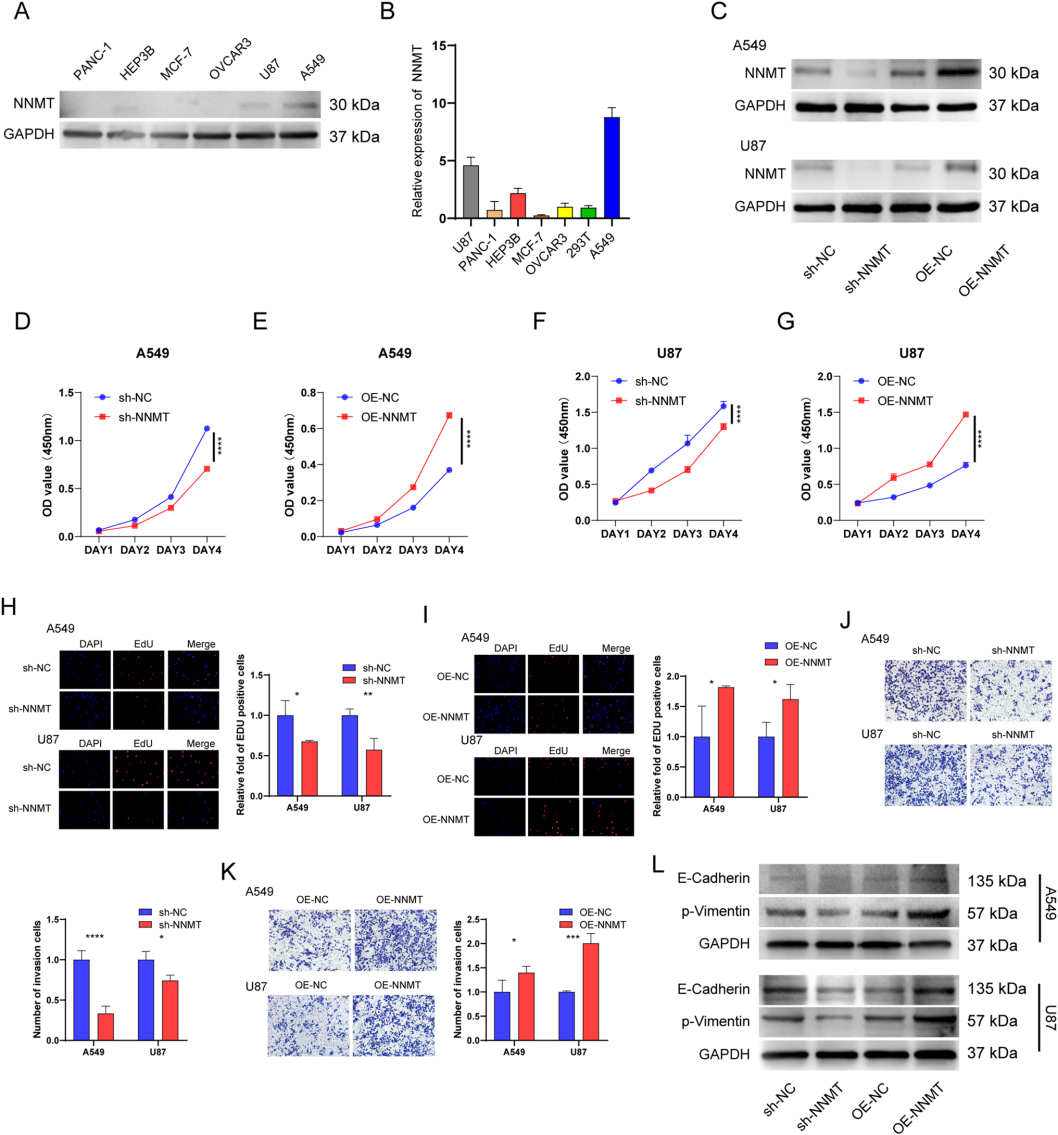
NNMT Regulation of Cancer Cell Proliferation and Migration. **A**, **B** Western blotting and qRT-PCR detecting NNMT expression in various cancer cell lines. **C** Efficiency of NNMT overexpression and knockdown in A549 and U87 cells measured by western blotting. **D**–**G** CCK-8 assay assessing cell proliferation in NNMT knockdown or overexpression compared to control cells (two-sided t-tests). **H**, **I** EdU assay measuring cell proliferation in NNMT knockdown or overexpression compared to control cells (two-sided t-tests). **J**, **K** Transwell assay showing cell migration in NNMT knockdown or overexpression compared to control cells. **L** Western blot analysis of E-Cadherin and p-Vimentin in A549 and U87 cells, (two-sided t-tests; nsP > 0.05, P < 0.05, **P < 0.01, ***P < 0.001, ****P < 0.0001)

To elucidate the functional role of NNMT, we assessed NNMT expression levels in PANC1, Hep3B, MCF-7, OVCAR3, U87, A549, and 293 T cell lines using western blot analysis and qRT-PCR. Notably, U87 and A549 exhibited the highest NNMT expression (Fig. 8A, B). We effectively achieved NNMT overexpression and knockdown in A549 and U87 cells, as evidenced by Western blotting (Fig. 8C). Subsequently, CCK8 and EdU assays were performed to investigate the impact of NNMT on cell proliferation. The results demonstrated that NNMT knockdown significantly inhibited cell proliferation compared to the control group, whereas NNMT overexpression promoted it (Fig. 8D–I). Furthermore, by Transwell assay, NNMT overexpression increased cell migration, while NNMT knockdown inhibited migration (Fig. 8J, K). The expression changes of invasion related markers E-Cadherin and p-Vimentin were detected by Western Blot analysis, and the results were also consistent (Fig. 8L). These findings underscore the crucial role of NNMT in promoting lung cancer and glioma cell growth and migration.

## Discussion

The present study offers a comprehensive examination of the functional significance of NNMT as a potential therapeutic target in human cancers. Combining bioinformatics analysis and experimental validation, we have demonstrated that NNMT is overexpressed in multiple cancer types and is associated with poor prognosis. Additionally, our findings suggest that NNMT is involved in the tumor microenvironment and correlates with the expression of immune checkpoint genes. Inhibition of NNMT in cancer cells resulted in reduced cell proliferation and migration, further supporting its potential as a therapeutic target.

Our findings are consistent with prior research linking NNMT to cancer development and progression. Previous studies have reported elevated NNMT expression in colorectal and breast cancers, promoting tumor growth, and metastasis, respectively [9, 26, 27]. Our results complement these findings and underscore the broader implications of NNMT dysregulation in various cancer types. Moreover, our study's observations regarding NNMT's association with the tumor immune microenvironment align with studies implicating NNMT in immune modulation. The positive correlation between NNMT expression and immune checkpoint genes is intriguing, suggesting potential crosstalk between NNMT and immune checkpoint pathways. These findings raise the possibility that NNMT inhibition could enhance the response to immune checkpoint inhibitors, suggesting a rationale for combination therapies targeting both NNMT and immune checkpoints. The observed hypomethylation of NNMT in tumors presents intriguing implications for our understanding of cancer biology. NNMT plays a pivotal role in epigenetic gene regulation, particularly through methylation mechanisms, and its dysregulation has been implicated in various cellular processes, including metabolism. The link between NNMT, epigenetic modifications, and cellular metabolism underscores its significance in cancer development and progression. Further research is warranted to elucidate the precise mechanisms underlying NNMT dysregulation in cancer and to explore its therapeutic implications fully.

The experimental validation of NNMT inhibition in cancer cell lines further supports its potential as a therapeutic target. Our results demonstrate that NNMT knockdown significantly reduces cell proliferation and migration, aligning with studies investigating NNMT’s functional consequences in various cancer types. While our study provides promising evidence, certain limitations must be acknowledged. Primarily, in vitro experiments using cell lines may not fully reflect the complexity of the tumor microenvironment in vivo. Hence, future investigations with animal models and patient-derived xenografts are necessary for more clinically relevant validation. Additionally, the underlying molecular mechanisms by which NNMT exerts its effects in cancer remain incompletely understood, warranting further exploration. Moreover, it is essential to consider potential off-target effects and toxicity when targeting NNMT therapeutically. Developing specific and selective NNMT inhibitors will be crucial to minimize undesired side effects. Encouragingly, some studies have identified small-molecule inhibitors of NNMT with anti-cancer effects in lung cancer cells, presenting promising candidates for future preclinical and clinical investigations.

Furthermore, the clinical relevance of NNMT as a biomarker and therapeutic target in cancer requires further exploration. While previous studies have linked NNMT expression to poor prognosis, the evaluation of NNMT as a predictive biomarker for treatment response and patient stratification is still lacking. Investigating the correlation between NNMT expression and clinical outcomes in larger patient cohorts and exploring its potential as a predictive biomarker for specific therapeutic interventions, including immunotherapies, is crucial. Moreover, considering the potential interplay between NNMT and other molecular pathways involved in cancer progression, such as the PI3K/Akt and Wnt/β-catenin pathways, will be essential for understanding NNMT-mediated oncogenesis and developing potential combinatorial therapeutic strategies.

In conclusion, our study provides compelling evidence supporting the functional significance of NNMT as a potential therapeutic target in human cancers. Its overexpression in various cancer types, association with poor prognosis, and impact on cellular phenotypes suggest its potential as a biomarker and therapeutic target. However, further research is required to elucidate the underlying mechanisms, assess the clinical implications, and evaluate the safety and efficacy of targeting NNMT in cancer treatment. Nevertheless, our findings lay the groundwork for future investigations and hold promise for the development of novel therapeutic strategies in oncology.

## Data Availability

Data from the TCGA was utilized and examined in the present investigation. For additional information on this study, please contact the corresponding author. The entire data needed to evaluate the findings can be found within this article.

## Abbreviations

OV: Ovarian serous cystadenocarcinoma
UCEC: Uterine corpus endometrial carcinoma
LGG: Brain lower grade glioma
HGG: Brain higher grade glioma
RCC: Renal cell carcinoma
LUSC: Lung squamous cell carcinoma
BLCA: Bladder urothelial carcinoma
COAD: Colon adenocarcinoma
CECS: Cervical squamous cell carcinoma
PCPG: Pheochromocytoma and paraganglioma
BLCA: Bladder urothelial carcinoma
BRCA: Breast invasive carcinoma
CHOL: Cholangiocarcinoma
COAD: Colon adenocarcinoma
ESCA: Esophageal carcinoma
GBM: Glioblastoma multiforme
HNSC: Head and neck cancer
KIRC: Kidney renal clear cell carcinoma
KIRP: Kidney renal papillary cell carcinoma
LIHC: Liver hepatocellular carcinoma
LUAD: Lung adenocarcinoma
LUSC: Lung squamous cell carcinoma
PRAD: Prostate adenocarcinoma
READ: Rectum adenocarcinoma
STAD: Stomach adenocarcinoma
THCA: Thyroid carcinoma
ACC: Adrenocortical carcinoma
CESC: Cervical squamous cell carcinoma and endocervical adenocarcinoma
LAML: Acute myeloid leukemia
PAAD: Pancreatic adenocarcinoma
SKCM: Skin cutaneous melanoma
TGCT: Testicular germ cell tumors
UCS: Uterine carcinosarcoma
